# Adipocytes in hematopoiesis and acute leukemia: friends, enemies, or innocent bystanders?

**DOI:** 10.1038/s41375-020-0886-x

**Published:** 2020-05-30

**Authors:** Julia Zinngrebe, Klaus-Michael Debatin, Pamela Fischer-Posovszky

**Affiliations:** Department of Pediatrics and Adolescent Medicine, Ulm University Medical Centre, D-89075 Ulm, Germany

**Keywords:** Preclinical research, Oncogenesis, Translational research

## Abstract

The bone marrow is home to well-balanced normal hematopoiesis, but also the stage of leukemia’s crime. Marrow adipose tissue (MAT) is a unique and versatile component of the bone marrow niche. While the importance of MAT for bone health has long been recognized, its complex role in hematopoiesis has only recently gained attention. In this review article we summarize recent conceptual advances in the field of MAT research and how these developments impact our understanding of MAT regulation of hematopoiesis. Elucidating routes of interaction and regulation between MAT and cells of the hematopoietic system are essential to pinpoint vulnerable processes resulting in malignant transformation. The concept of white adipose tissue contributing to cancer development and progression on the cellular, metabolic, and systemic level is generally accepted. The role of MAT in malignant hematopoiesis, however, is controversial. MAT is very sensitive to changes in the patient’s metabolic status hampering a clear definition of its role in different clinical situations. Here, we discuss future directions for leukemia research in the context of metabolism-induced modifications of MAT and other adipose tissues and how this might impact on leukemia cell survival, proliferation, and antileukemic therapy.

## Introduction

The bone marrow microenvironment (BMM) is characterized by a complex network of different cell types providing key signaling events for the regulation of hematopoiesis. Although leukemia is caused by a series of genetic hits to hematopoietic stem and progenitor cells leading to malignant transformation [1–3], evidence is accumulating that the BMM also influences development and progression of malignancies in the bone marrow (BM). Alterations in niche cells may also contribute to leukemogenesis. For example, deletion of *Dicer1*, the key factor of microRNA maturation in osteoprogenitors, but not in mature osteoblasts, led to disruption of hematopoietic integrity and resulted in myelodysplasia and emergence of acute myelogenous leukemia (AML) in mice [4]. Furthermore, the local environment in the BM is also crucial for leukemia cell survival and might play a role in resistance to chemotherapy and disease relapse. A prominent example for interaction of leukemic cells with their surroundings is the CXC motif ligand 12 and CXC chemokine receptor 4 (CXCL12/CXCR4) axis [5]. The chemokine CXCL12 is secreted by BM stromal cells and serves as a potent chemo-attractant for CXCR4-positive hematopoietic cells, withholding them in an environment equipped with growth factors essential for their survival. This mechanism can be hijacked by AML cells, which also express CXCR4 [6]. On these grounds, the concept of inhibition of the leukemia–niche interaction was developed as novel therapeutic strategy. Indeed, targeting the CXCL12/CXCR4 axis appears to deliver the promising results in early-phase clinical trials [6], and it is expected that additional routes of interaction between leukemia and the BMM will be discovered. Interestingly, a prominent cell type within the BMM has long been neglected and only recently entered center stage: the BM adipocyte. Adipocytes are highly active secretory cells. Via their secretion products, which are collectively referred to as adipokines, they contribute to the development and progression of solid tumors, which are surrounded by adipose tissue (AT) [7]. There is accumulating evidence that they might play a similar role in the context of leukemia in the local BMM but also systemically *via* endocrine routes of interaction.

### The colors of fat—white, brown, beige, pink, and yellow

**Fig. 1:**
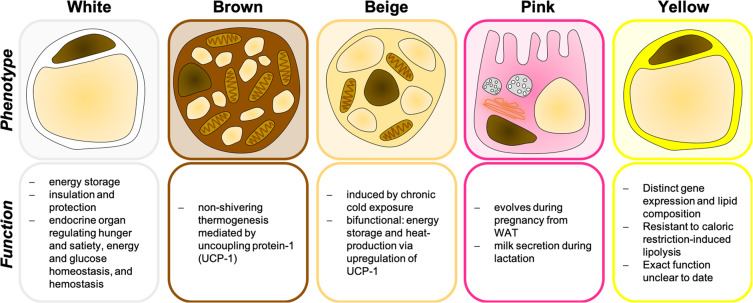
The colors of fat—white, brown, beige, pink, and yellow. The typical phenotypic appearance of white, brown, beige, pink, and yellow adipocytes is depicted with the distinct functions of the respective adipose tissue.

When talking about AT, we usually refer to the two classical types, i.e., white adipose tissue (WAT) and brown adipose tissue (BAT) (Fig. 1). WAT covers the human body in a subcutaneous layer but also surrounds the inner organs in visceral depots, thereby providing insulation and protection [8]. It serves as an endocrine organ regulating processes as important as hunger and satiety, energy and glucose homeostasis, and hemostasis but its best-known function is storage of surplus energy in form of triglycerides, which can again be released in case of higher demand or food shortage [8].

BAT, instead of saving fat, has the unique capability of using up stored triglycerides for production of heat in a process called nonshivering thermogenesis [9]. This process is mediated by uncoupling protein-1 (UCP1), which is located at the inner mitochondrial membrane of brown adipocytes and, *nomen est omen*, uncouples the oxidative phosphorylation from the ATP-synthase to generate heat instead of ATP [10]. The brown color results from high amounts of cytochrome C in this mitochondria-rich tissue.

Beige (brite, inducible BAT-like) adipocytes can arise within WAT depots upon chronic cold exposure (reviewed in [11]) (Fig. 1). They represent an intermediate, bifunctional phenotype of adipocytes, which stores triglycerides and also contributes to heat production via upregulation of UCP1 [11]. In rodents, beige AT is responsible for the maintenance of body temperature during cold temperatures [11]. Pink AT evolves during pregnancy and lactation from WAT via transdifferentiation, consists of milk-secreting alveolar cells and appears pink in color (review in [12]) (Fig. 1). Among these four types, only WAT has been linked to extramedullary hematopoiesis as well as leukemia cell biology as described below.

The color yellow was assigned to marrow adipose tissue (MAT) (Fig. 1). In a healthy, lean person, MAT represents over 10% of total fat mass [13]. Although adipocytes of the BM resemble the white adipocyte’s unilocular morphology, they display a distinct gene expression [14], lipid profile [14, 15] and metabolism, e.g., seem to be resistant to calorie restriction-induced hydrolysis of stored triglycerides [15].

### Our bones are full of fat—but why?

**Fig. 2:**
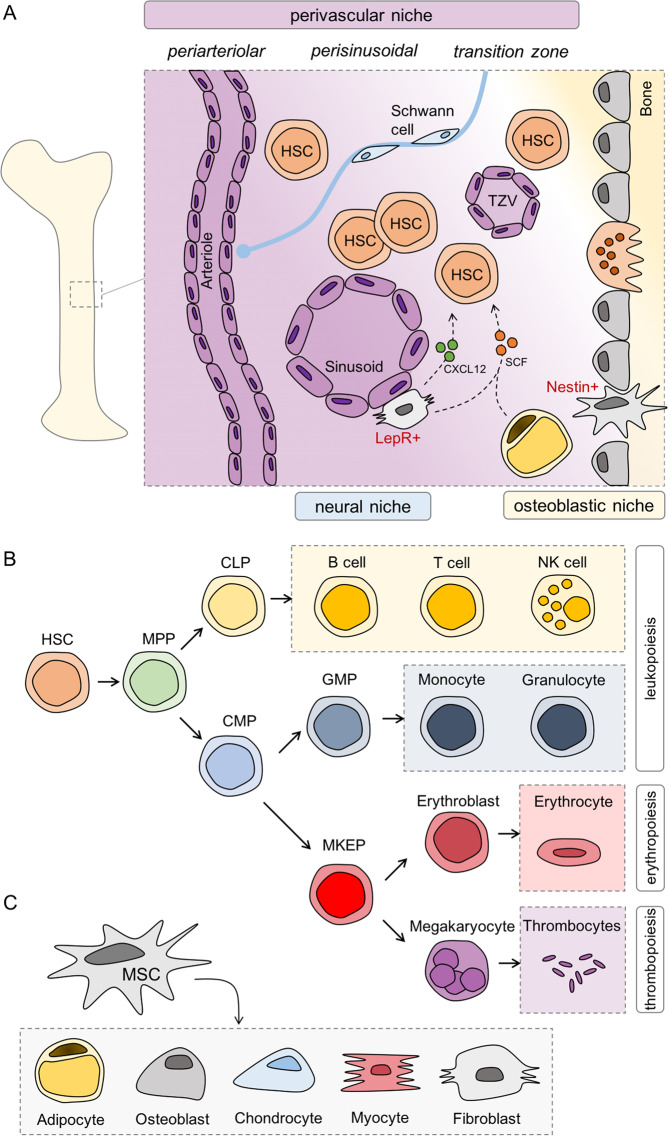
Compartments and components of the bone marrow microenvironment (BMM). **a** The bone cavity is formed by osteoblasts and contains the bone marrow (BM) with its microenvironment. In this cavity, hematopoiesis is maintained by hematopoietic stem cells (HSCs). HSCs are mainly found in the perivascular niche which can further be divided into periarteriolar (around arterioles), perisinusoidal (around sinusoids), and transition zone (around transition zone vessels). The osteoblastic niche is located near the endosteum and contains only a very small number of HSCs. The BM is innervated by nerve fibers reaching to stromal cells and pericytes (not depicted) around arterioles forming the neural niche and regulating HSC fate. **b** HSCs can further differentiate into common lymphoid progenitors (CLPs), the common progenitor of B cells, T cells, or NK cells, or common myeloid progenitors (CMPs) which further differentiate into progenitors of either erythrocytes and megakaryocytes or monocytes and granulocytes. **c** Mesenchymal stem cells (MSCs) are multipotent stem cells which can differentiate into different kinds of cells, including adipocytes, osteoblasts, chondrocytes, myocytes, or fibroblasts.

The bone cavity formed by osteoblasts harbors the BM and is home to hematopoiesis (Fig. 2a) maintained by hematopoietic stem cells (HSCs). HSCs have two essential features: they possess (1) self-renewal activity and (2) multipotency, i.e., they are able to differentiate into all mature blood cells [16]. Common myeloid progenitors or common lymphoid progenitors give rise to cells from either the myeloid lineage, which include monocytes, granulocytes, erythrocytes, and platelets or from the lymphoid lineage, i.e., B cells, T cells, or NK cells, respectively (Fig. 2b). The BM comprises MSCs that can further differentiate into other cell types, e.g., adipocytes, osteoblasts, chondrocytes, and myocytes [17] but also fibroblasts [18] and components of the nervous system [19] (Fig. 2c). Whether MSCs can also give rise to endothelial cells is still controversially discussed [20].

Recently, the murine BM was analyzed by single-cell RNA-sequencing (scRNA-seq) either under normal or stressed conditions [21] or in the context of AML [22]. Both studies report a dynamic cellular heterogeneity of the niche and an impressive plasticity in response to exogenous stimuli.

HSCs critically depend on factors provided by the BMM such as stem cell factor (SCF) and CXCL12 [23–25]. Perivascular stromal cells, also known as CAR (CXCL12-abundant reticular) cells, are the main source of CXCL12 [26], but it is also expressed in endothelial cells and osteoblasts, albeit at lower levels [24]. Perivascular stromal cells and endothelial cells are crucial components of the HSC niche, which is highlighted by the fact that both cell types secrete SCF [24]. Moreover, BM adipocytes are also a major supplier of SCF [27] and a study in mice with an adipocyte-specific knockout revealed that MAT-derived SCF is essential for hematopoiesis both under steady state and metabolic stress conditions [28].

HSCs reside in special “niches” in which their self-renewal and differentiation are influenced by signals from the surrounding BMM, a concept dating back to 1978 [29]. Most HSCs reside close to vessels in the BM, i.e., 80% of HSCs around sinusoidal vessels (perisinusoidal), 10% around arterioles (periarteriolar), and another 10% close to transition zone vessels [30]. Only a very small number of HSCs is located near the endosteum [29, 31] and osteoblasts are not required for HSC maintenance [32]. Thus, the previously proposed model of an osteoblastic or endosteal HSC niche [33, 34] appears to be outdated [35].

In utero and at the time of birth, the bone cavities are predominantly occupied by hematopoietic red marrow [36]. The formation of MAT starts during childhood and it further increases with age in a defined spatio–temporal manner from distal to proximal (for review see [37]). By the age of 25 years ~50–70% of BM space is already occupied by adipocytes [37]. It was proposed that adipocytes in the BM are adjacent to the endosteal surface in the metaphysis [38]. More recent work shows that adipocytes are in close vicinity to the sinusoidal vasculature and sympathetically innervated [39]. In addition, adipocytes are in contact with a subset of hematopoietic cells, e.g., cells of the myeloid and granulocyte lineage as well as erythroblast islands [39].

The existence of two different types of MAT was suggested already in 1976 [40]. Recently, this concept has been brought back to life by Scheller et al. proposing the concept of regulated MAT (rMAT) and constitutive MAT (cMAT) [41]. They demonstrated that rMAT presents as single adipocytes and is located in more proximal regions at sites of active hematopoiesis, whereas cMAT is found in distal bones with low numbers of hematopoietic cells interspersed within adipocytes [41]. During childhood, cMAT develops earlier than rMAT [37] is more resilient to environmental and metabolic changes, and contains larger adipocytes, which differ in their gene expression and lipid composition from rMAT adipocytes [41].

For decades MAT was considered a mere filler of the otherwise empty BM space and we have just begun to understand that its function goes far beyond. Comparable to WAT, MAT was identified as an endocrine organ [13]. BM adipocytes secrete leptin [42], a hormone involved in the central nervous regulation of body weight, puberty, and fertility [43], and adiponectin, a regulator of metabolism and systemic insulin sensitivity [13]. Therefore, MAT might serve as an “integrator” of the systemic nutritional and metabolic state into hematopoiesis.

Interestingly, MAT increases under certain clinical conditions including osteoporosis [44, 45], ageing [41, 44], metabolic diseases such as obesity [46] and diabetes (reviewed in [47]), and, counterintuitively, also in patients with anorexia nervosa [48] and under caloric restriction [49], whereas exercise can lower the MAT volume [50]. MAT also expands during therapeutic interventions, e.g., radiation [51], chemotherapy [52], and treatment with glucocorticoids [52, 53]. A massive decrease or loss of MAT seems to occur only under severe energy deprivation as observed in patients with late stage anorexia nervosa, leading to gelatinous transformation of the BM [54].

Taken together, MAT is characterized by a dynamic plasticity and region-specific differences. The large variety of influencing factors seem to hamper systematic investigations and may account for controversies found in the literature.

### MAT in normal hematopoiesis

We are only beginning to understand the role of MAT in normal hematopoiesis. In a landmark paper, Naveiras et al. identified MAT as negative regulator of hematopoiesis [55]. They showed that adipocyte-rich BM isolated from tail vertebrae had reduced numbers and frequencies of HSCs and short-term progenitors along with an impaired cycling capacity as compared with adipocyte-free thoracic vertebrae [55]. To corroborate an inhibitory role of MAT on hematopoiesis in the BMM, a pharmacological approach to inhibit adipogenesis as well as a genetic approach using the “fatless” A-ZIP/F1 mouse model was chosen. Under both conditions, BM engraftment after irradiation was accelerated [55], and the similar results were obtained by another group [56]. In addition, an increase in MAT observed during obesity or aging impaired hematopoiesis [57]. Remarkably though, MAT seems to differentially regulate the function of short-term and long-term HSCs because HSCs isolated from adipocyte-rich regions showed a better long-term engraftment [56]. This interesting finding was substantiated in a co-culture study of HSCs with primary human MSCs or MSC-derived adipocytes from the femoral head of patients undergoing hip surgery [58]. Whereas HSCs were stably maintained during long-term ex vivo culture with MSCs, adipocytes first induced a decline but later again an increase in colony-forming units suggesting that BM-derived adipocytes may enhance long-term maintenance of HSCs [58].

### MAT in malignant hematopoiesis

There is accumulating evidence that MAT also influences survival and proliferation of leukemic blasts. Decline of normal hematopoiesis during leukemia progression is a well-known phenomenon, but has only recently been attributed to a disturbance of MAT function by AML cells resulting in compromised myelo-erythropoiesis [59]. In contrast to MAT's supportive function of normal hematopoiesis during leukemia, its role in leukemia development is controversial and might be lineage specific. Thus, MAT was found to negatively affect T-ALL proliferation in vitro and in vivo thereby mediating chemoresistance [60]. On the other hand, MAT supported the survival and proliferation of AML blasts from patients [61] by hijacking the metabolism of adipocytes to induce free fatty acid release via activation of lipolysis [61]. Mechanistically, fatty acid binding protein-4 (FABP4) was essential for proliferation of AML blasts in co-culture with adipocytes as its knockdown by shRNA prevented AML blast proliferation, and, in addition, increased survival of mice in a model of Hoxa9/Meis1-driven AML [61].

Caloric restriction is known to result in an increase of MAT [49]. Interestingly, fasting or dietary restrictions improve the outcome of different cancer entities. A recent paper by Lu et al. demonstrates that this also applies to B-ALL and T-ALL, but not AML [62]. Repeated cycles of 1 day fasting followed by 1 day of feeding shortly after transplantation of leukemia cells completely inhibited leukemia development in the N-Myc B-ALL model and massively decreased the leukemic burden in the Notch1 T-ALL model. Mechanistically, fasting led to an upregulation of *Lepr* expression and activation of the downstream signaling molecule PR/SET domain 1 in leukemic cells [62]. In contrast, the dietary regime had no beneficial effect in the MLL-AF9 AML model. The authors speculate that AML cells have high basal expression levels of *Lepr* and might therefore be resistant to fasting-induced upregulation of this pathway. In ALL, fasting not only prevented leukemia development, but also resulted in differentiation and depletion of leukemic cells. Most importantly though, fasting also effectively inhibited the growth of human patient-derived B-ALL in a xenograft model and the expression of LEPR signaling-related genes was correlated to prognosis in pediatric pre-B-ALL [62]. Clearly, these findings open up new therapeutic strategies for the treatment of ALL. It can be speculated that other fasting-responsive pathways are also major contributors, e.g., the IGF-1/IGF-1R growth hormone axis [62]. The concept of treating leukemia by forcing terminal differentiation of the malignant cell instead of killing it was proposed 40 years ago (for review see [63]). The findings by Lu et al. encourage the revival of this old concept from a new perspective: “starving leukemia to induce differentiation” [64].

### Influence of other fat depots on normal and malignant hematopoiesis

Back in 1962, infiltrations of leukemia or lymphoma cells were identified in subcutaneous AT of patients [65]. In mice, HSCs are not only found in the BM, but also in extramedullary gonadal WAT (gWAT) [66]. gWAT represents the largest visceral fat depot in mice and was just recently identified as a reservoir of leukemic stem cells (LSCs) in a model of blast crisis chronic myeloid leukemia [67]. The LSCs present in gWAT had a proinflammatory gene signature and induced lipolysis leading to atrophy of AT. Interestingly, fatty acid oxidation (FAO) was increased in leukemic versus nonleukemic cells, and LSCs presented with the highest FAO rate suggesting that these cells might benefit most from lipolysis. In line, the fatty acid transporter CD36 was highly, but not uniformly expressed in LSCs. Specifically, CD36 + LSCs were strikingly enriched in gWAT and were protected from chemotherapy by the local AT microenvironment. Vice versa, the knockout of CD36 decreased the leukemic burden in gWAT and sensitized LSCs to chemotherapy. Thus, LSCs may hide in extramedullary AT, metabolically adapt to this local environment by shifting their metabolism toward fatty acid uptake and oxidation and thereby escape chemotherapy. Targeting fatty acid metabolism not only on the leukemia side but also in the niche might therefore provide an interesting strategy to eradicate LSCs.

### Protection of leukemic cells from chemotherapy-induced cytotoxicity by adipocytes?

Evidence is accumulating that leukemic cells, when in close proximity to adipocytes, are protected from chemotherapy-induced cytotoxicity. Mechanisms by which adipocytes might provide shelter to leukemic blasts were intensively studied by Mittelman et al. in both, in vitro and in vivo models [68–73]. Murine 8093 pre-B-ALL cells were transplanted into male C57BL/6 mice, which were either obese due to a high fat diet (HFD) or had been fed a normal diet [69]. Obesity did not influence the time of onset of progressive leukemia but it impaired the treatment effect of vincristine [69] supported by earlier studies showing that vincristine pharmacokinetics can be influenced by HFD-induced obesity in C57BL/6 mice [68]. In this study, the applied vincristine dose was adjusted to total body weight. The obese animals received ~28% more vincristine in total and had comparable levels of the drug in both, circulation and tissues. Thus, the authors concluded that the impaired antileukemic effect was not due to altered vincristine exposure [69]. Most interestingly, numerous leukemia cells were detected in WAT [69] in different locations [71] in the mouse models, possibly attracted by SDF1a [71] suggesting that leukemia cells might hide from chemotherapy in the fat pads. In line, co-culture with adipocytes not only deteriorated the cytotoxic effect of vincristine but also of dexamethasone, daunorubicin, or nilotinib in murine and also human ALL cell lines (SD-1, RS4; 11 and BV173) [69]. Mechanistically, this observation was attributed to an upregulation of the antiapoptotic proteins, B-cell lymphoma 2 and serine/threonine-protein kinase pim-2 by adipocyte co-culture in leukemia cells [69].

The same syngeneic HFD-ALL mouse model was used for the analysis of the response to l-asparaginase (ASNase) [70], a first-line drug for ALL treatment which degrades asparagine and glutamine, amino acids essential for the survival of leukemic cells. Adipocytes are an important source of systemic glutamine [74] and it is well conceivable that high local levels of this amino acid protect from ASNase-induced toxicity. Indeed, comparable to the above described study [69], obesity significantly reduced survival in response to treatment with ASNase-treated mice compared with lean animals [70]. Via secretion of glutamine adipocytes also reduced the antileukemic effect of ASNase in vitro. This is in line with the finding that expression of glutamine synthetase was increased in BM sections of adolescent patients with leukemia upon induction therapy, yet the plasma levels of asparagine and glutamine are comparable between lean and obese children undergoing high-risk ALL treatment [70].

**Fig. 3:**
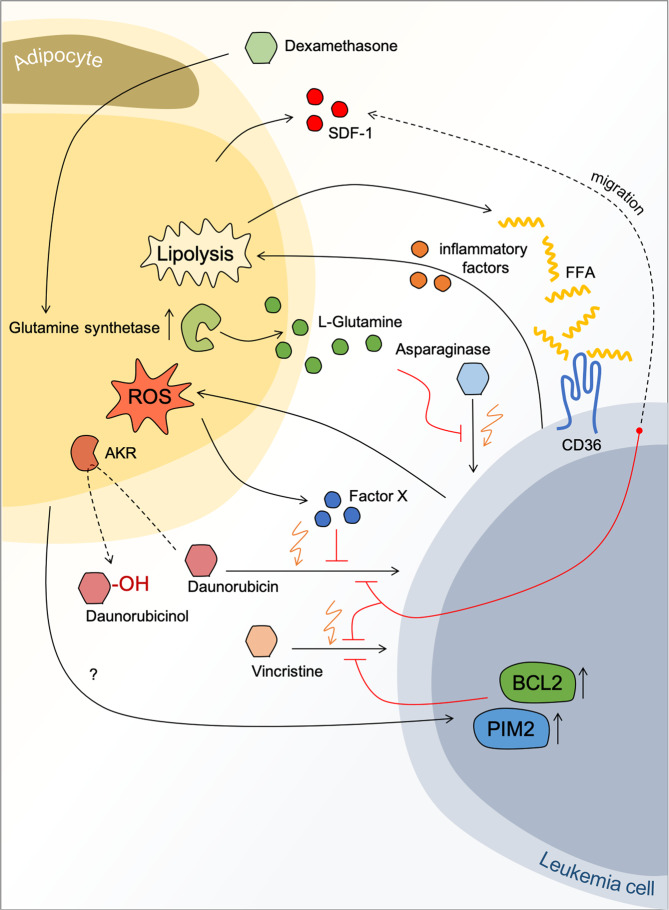
The influence of adipocytes on antileukemic therapy. Depicted are different ways of how adipocytes were shown to interfere with antileukemic therapy.

Most importantly, WAT is not only able to attract leukemia cells via the secretion of chemotactic factors [71] but seems to take up and metabolize chemotherapeutic drugs. Adipocytes are characterized by high expression of enzymes capable to convert Daunorubicin to the less effective Daunorubicinol, i.e., aldo–keto reductases (AKR) and carbonyl reductases [73]. AKR activity was detectable in AT explants and 3T3-L1-derived adipocytes. In the presence of adipocytes, there is significantly less accumulation of Daunorubicin in ALL cells as adipocytes can absorb and metabolize anthracyclines [73]. In addition to altering pharmacodynamics, adipocytes might also give protection to leukemia cells by modulating the oxidative stress response [72]. Cell–cell contact with ALL cells or incubation with ALL-conditioned media causes oxidative stress in adipocytes which in turn leads to secretion of yet unknown factors, which mediate protection from Daunorubicin-induced cell death [72]. A summary of the known aspects on how adipocytes influence response of leukemia cells to chemotherapy is illustrated in Fig. 3.

### Disrupting adipocyte–leukemic cell interaction as novel therapeutic target

**Fig. 4:**
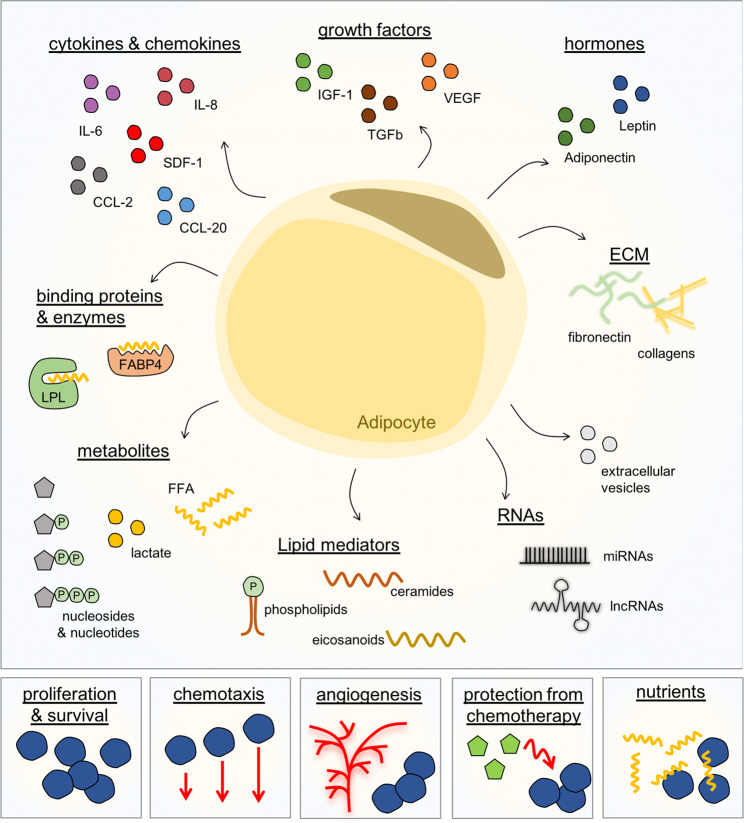
Disrupting adipocyte–leukemic cell interaction as novel therapeutic target. Adipocytes secrete many different factors, amongst them classical hormones, growth factors, cytokines, and chemokines, components of the extracellular matrix (ECM), binding proteins or enzymes, different types of metabolites, but also microRNAs and long noncoding RNAs or extracellular vesicles. These different classes of factors might influence proliferation and survival of leukemia cells directly or indirectly via different mechanisms.

The concept of targeting the interaction of leukemia cells with their specific niche in the BM has emerged only recently [38, 75] and adipocytes should definitively be added to the list of potential target structures. Adipocytes are highly active secretory cells and release a large variety of factors to the local environment and the circulation [76] (Fig. 4). These comprise proteins, among them classical hormones such as leptin or adiponectin, growth factors, e.g., insulin-like growth factor-1 (IGF-1), vascular endothelial growth factor (VEGF), transforming growth factor-beta (TGFβ), cytokines and chemokines, e.g., interleukin-6 (IL-6), interleukin-8 (IL-8), CC-chemokine ligand 2 (CCL-2), CC-chemokine ligand 20 (CCL-20), SDF-1, components of the extracellular matrix, e.g., fibronectin or collagens, but also binding proteins or enzymes important for the uptake of fatty acids, e.g., FABP4 or lipoprotein lipase. They also include different types of metabolites such as fatty acids, lactate or nucleosides and nucleotides. Especially extracellular ATP (eATP) seems to be of interest in the context of normal and malignant hematopoiesis as eATP has been implicated in mobilization of HSCs from BM to peripheral blood [77, 78] but also interfered with proliferation and homing of AML blasts [79]. Adipocytes also release lipid mediators, e.g., ceramides or eicosanoids such as prostaglandins and leukotrienes, but also bioactive phospholipids. The latter include sphingosine-1-phosphate (S1P) and ceramide-1-phosphate (C1P), which have been identified as potent chemoattractants for HSCs [80, 81] and leukemic cells [82]. Thus, S1P and C1P might also play an important role in migration and homing of leukemic cells into AT. In addition, microRNAs and long noncoding RNAs or extracellular vesicles are also factors secreted by adipocytes [76]. All these different classes of factors might be present in the local microenvironment and influence leukemia cells directly via cell–cell contact or in a paracrine manner, but also via impacting other cell types present in the BMM. Mechanisms of action include stimulation of proliferation and survival of leukemia cells, maintaining leukemia cells in the niche by an inviting matrix and/or a chemotactic gradient, modulation of the vascular niche/angiogenesis in the BM, protection from chemotherapy and well conceivably, delivery of nutrients, and alteration of leukemia cell gene expression or metabolism (Fig. 4). Aging leads to a massive increase in MAT and age is a major risk factor for leukemia. Whether adipocytes, similar to osteoprogenitors [4], can contribute to the development and onset of leukemia in the BMM is unclear at this point.

Recent scRNA-seq studies on murine BM stroma identified factors with specific relevance for leukemia treatment. For example, the compartment of leptin receptor-positive (Lepr^+^) cells was analyzed, which contained four different clusters of which two expressed adipogenesis-associated marker genes [21]. Interestingly, these two adipocytic-primed clusters of Lepr^+^ cells were identified as major source of prohematopoietic factors in the BMM, amongst them SDF-1 (*Cxcl12*), SCF (*Kitl*), IL-7 (*Il7*), IL-15 (*Il15*), IL-34 (Il-34), M-CSF (Csf1), BMP-4 (Bmp4), CCL-19 (Ccl19), and CCL-2 (Ccl-2) [21]. Gene expression analyses on human samples confirmed an enrichment of inflammatory genes in BM adipocytes [83]. Several of these factors are already exploited for the treatment of solid tumors and/or leukemia or are in preclinical testing and clinical trials (for review see [84]). Whether the mode of action of novel compounds, e.g., neutralizing antibodies involves adipocytes or other cell types as source of inflammatory factors in the local niche is not clear at this point. Also, whether modulating the adipocyte compartment in the BM may serve as a sensitizing strategy requires further study. Based on the major part of the recent literature adipocytes provide a protective environment, acting as “friends” of leukemia cells. Thus, eliminating adipocytes from the BMM might help to eliminate leukemic cells. However, getting rid of BM adipocytes is a complicated task as there is only a very limited number of factors reducing MAT including parathyroid hormone [85], the diabetes medication metformin [86], mechanical loading [87], and exercise [88]. One study by Liu et al. reports that chemotherapy leads to an inhibition of adipogenesis in the BM via mononuclear cell-derived growth differentiation factor 15 (GDF-15), in turn reinforcing the efficacy of consolidation chemotherapy in AML patients during complete remission [89].

Fasting, a condition known to enhance MAT, inhibited the engraftment and progression of B-ALL and T-ALL in several mouse model systems tested [62]. From this finding, one might conclude that adipocytes are “enemies” of leukemia cells in the BMM. Lu et al. did not comment on BM adipocytes in their study but they report that, as expected, fasting induced a drop in circulating and local leptin levels [62]. Leptin is known to inhibit adipogenesis in the BM in vitro and in vivo [90, 91], thus we can expect an increase in MAT with low levels of leptin. Most likely due to low leptin, they identified an upregulation of *Lepr* expression in leukemia cells and activation of downstream signaling routes as main cause leading to inhibition of leukemia initiation and progression. *Vice versa*, attenuated *Lepr* expression was identified as essential for development and maintenance of ALL [62]. All in all, the leptin/Lepr axis is an interesting new target for leukemia treatment.

Besides acting as a member of the local BMM, adipocytes can certainly also interact with leukemia cells via their secreted factors in an endocrine manner. This assumption is underlined by the fact that obesity, which is characterized by the excessive accumulation of WAT, leads to an increased risk of developing and dying from leukemia as recently revealed in a meta-analysis of prospective cohort studies [92]. The fact that leukemia cells use WAT as a hideaway to escape chemotherapy should also be taken into account. Therefore, not only strategies to reduce MAT but also WAT might be useful to support leukemia treatment and also maintain remission.

### Open questions and future directions of scientific research

Fasting is currently propagated as the holy grail of cancer treatment by some researchers [93]. Since a complete abstinence from all food and drinks except water is hard to achieve, specific fasting-mimicking diets (FMDs) were developed. These diets are designed to mimic the beneficial effects of fasting. They include proteins and carbohydrates and make up a daily energy intake of 300–1000 kcal. They have reduced nutritional risk compared with complete abstinence and a much better patient compliance (for review see [93]). The dietary regime is applied in cycles, e.g., 1–5 days of fasting every 3–4 weeks [93]. Intensive research is urgently needed in this area to determine long-term outcome and sequelae of such interventions [94, 95]. The first results seem very promising as fasting or FMDs show robust anticancer effects in animal models and also in clinical trials [93], yet the effect on hematological disease is largely unexplored. Fasting interventions have an impact on systemic metabolism, basically switching the body into a mode of making use of its energy stores, which are mainly represented by WAT. The hallmark of the fasting response includes low levels of glucose and insulin, high levels of glucagon and ketone bodies, low levels of leptin and IGF-1 and high levels of adiponectin (for review see [93]). In normal cells, the altered circulating levels of metabolites and hormones will lead to a shutdown of metabolic activity and cell cycling, resulting in a protection from chemotherapeutic insults. In contrast, malignant cells can show the opposite response and are therefore sensitized to chemotherapy and also other therapeutic options.

It is very obvious that the key alterations induced by fasting are related to altered function of AT. A decrease in leptin and a subsequent upregulation of *Lepr* was already identified as protective mechanism in B-ALL and T-ALL development [62]. It will be important to identify other adipocyte-secreted factors that contribute to the beneficial effects of fasting in cancer therapy in general and in leukemia in particular. Leukemia develops in the BM. Thus, the effect of fasting on BM adipocytes needs to be dissected in detail. For example, a recent paper by Attané et al. described that BM adipocytes display distinct metabolic characteristics with an absence of lipolytic activity and a shift toward a cholesterol-oriented metabolism [15]. How this impacts on the interaction between BM adipocytes and leukemia needs to be investigated. It is also important to find out why fasting seems to be beneficial in ALL, but not AML. This might be related to different metabolic demands and signaling capacities of lymphoid and myeloid cells. Most importantly, we should aim at investigating whether fasting itself is required or if the fasting procedure can be mimicked by pharmacological agents, the polyphenolic compound resveratrol (RSV) being a prominent example [96]. RSV inhibits adipogenic differentiation and the expression of proinflammatory factors from adipocytes [97]. At the same time, RSV sensitizes leukemia cells to apoptosis induction [98]. It is conceivable that RSV also influences the interaction of both cell types in the local niche. Metformin is another promising candidate in this regard—it molecularly mimics caloric restriction [99], reduces the cancer incidence in both mice and humans [100] and, of note, reduces MAT [86]. By the way, comparable considerations may also apply to exercise. Physical exercise is beneficial during and after cancer treatment [101] and leads to the loss of WAT mass [102] and, importantly, also MAT [88].

Another important finding that requires appropriate follow-up is the accumulation of leukemia cells in WAT found in several mouse models [67, 68, 71] but also in humans [65]. In this old study dating back to 1962, WAT samples of 60 patients who died of leukemia or lymphoma were analyzed. 19 of them showed subcutaneous WAT infiltrations of malignant cells in apparently normal skin [65]. This is a remarkable observation, given the fact that WAT was commonly regarded as negligible organ in leukemia. WAT seems to serve as a hideaway for leukemic cells and supports evasion from chemotherapy as adipocytes are able to provide a protective environment, in part by metabolizing chemotherapeutic drugs [67–73]. Therefore, the accumulation of WAT in obesity hampers chemotherapy [68, 69, 73]. In animal studies, the dosage of chemotherapeutics used was adjusted to body weight. Of note, although obese animals received ~28% more vincristine, they had comparable levels in the circulation and in tissues [69]. The question arising from this observation is whether patients with an increased fat mass are actually underdosed. The body surface area is commonly used to calculate dosages for chemotherapeutic drugs and the commonly used formulas fit fairly well also in overweight and obese patients [103]. Nevertheless, altered pharmacodynamics in WAT should be taken into account, also in postremission therapy. This is underlined by the fact that obesity is associated with poorer outcome in several types of leukemia, including acute promyelocytic leukemia, AML, and pediatric pre-B-ALL [104–106].

### Summary

Taken together, although neglected in the past, MAT as well as WAT should be considered important and relevant in the context of leukemia. Their secretion products such as growth factors, cytokines, chemokines, matrix molecules, or metabolites have an impact on the function of leukemic cells opening up new therapeutic opportunities to fight hematological malignancy.
